# Reply to reaction on ‘Organ donation after euthanasia starting at home in a patient with multiple system atrophy – case report’

**DOI:** 10.1186/s12910-023-00914-z

**Published:** 2023-05-29

**Authors:** Najat Tajaâte, Nathalie van Dijk, Elien Pragt, David Shaw, A Kempener-Deguelle, Wim de Jongh, Jan Bollen, Walther van Mook

**Affiliations:** 1 Department of Anesthesiology, Zuyderland Medisch Centrum, Heerlen, The Netherlands; 2 Department of Intensive Care Medicine, Maastricht University Medical Center, Maastricht, The Netherlands; 3 Care and Public Health Research Institute, Maastricht University, Maastricht, the Netherlands; 4 Institute of Biomedical Ethics, University of Basel, Basel, Switzerland; 5 Department of Organ Donation Coordination, Department of Organ Donation Coordination, Maastricht, The Netherlands; 6 Department of Anesthesiology, Pain and Palliative Medicine, Radboud University Medical Center, PO Box 9101, 6500 HB Nijmegen, The Netherlands

**Keywords:** Euthanasia, Organ donation, Ethics, Medical law, Organ transplantation, Palliative sedation

## Abstract

We would like to respond to the comment we received from our colleagues on our case report about organ donation after euthanasia starting at home. We reply to their statements on medical and legal aspects, and provide more information on our view of informed consent.

Dear editor,

We would like to thank our colleagues Mulder and Sonneveld for their insightful reply [1]. In the Netherlands, more than 100 patients were able to donate their organs following euthanasia since 2012, of which four underwent an organ donation after euthanasia starting from home (ODEH) procedure. These patients did not want to close their eyes for the last time in a hospital, where they had already spent too much time during their life, but instead chose to fall asleep at home. They were sedated at home and transported by an ambulance to a hospital, where they underwent euthanasia and donated their organs.

Undeniably, this fully meets the principle of autonomy, as the patients are able to choose their own death (in detail). The current Dutch guideline on organ donation after euthanasia indeed states that patients preferably request this combined procedure themselves, to avoid any external pressure on the patient [2]. Mulder and Sonneveld stated that Canadian guidelines demand patient-initiated requests. However, the Canadian Blood Services has recommended that referral to the organ donation organization should occur as soon as is practical after the decision to proceed with withdrawal of life sustaining measures or determination of eligibility for medical assistance in dying (MAiD) [3]. We believe there can only be informed consent if the patient has been informed about *all* possibilities, including organ donation following MAiD. Preferably the physician only informs the patient about this procedure if they are medically eligible for donation – which is generally not the case if they suffer from malignancy. In the original case report, we stated that “patients are nevertheless willing to help others”. This had nothing to do with whether patients are actively asked to cooperate with organ donation or not. The patient in our case report requested organ donation himself, thus he was also ‘willing’ to help others.

We dispute that there are no legal concerns when performing ODEH. When the patient is sedated to be transported to the hospital, their suffering is relieved and the due diligence requirement of an unbearable and hopeless suffering is not fulfilled anymore. However, as the suffering will be present again when the sedatives are stopped, the review committees have found this procedure to be in accordance with the relevant legislation. The legal concern however is this: the Dutch guidelines on euthanasia state – very strictly – that only the physician who has verified the due diligence requirements is allowed to administer the euthanasia drugs, causing the patient’s death. The physician who sedates the patient (in our case an anesthesiologist) has not checked the due diligence criteria for euthanasia and does not have the type of treatment relationship with the patient that is required to perform euthanasia. If a medical complication during sedation, intubation or transport would occur, this might lead to a non-natural death and thus possible prosecution of the anesthesiologist. To avoid such legal problems, one could have the general practitioner perform the sedation (or at least administer the medication), but most general practitioners do not have any experience with this kind of sedation. It should be noted that euthanasia is always a non-natural death, and the review committee will have to decide whether the procedure was performed in line with the due diligence criteria.

Mulder and Sonneveld are absolutely right that the risk of awareness when using midazolam is dose dependent, which is why we also administered propofol (2 mg/kg/hr) – next to midazolam and piritramide in high doses - following intubation and during transport to the hospital. Since such state of the art patient treatment is at the core of each anesthesiologist’s performance, this was however not mentioned in our case report.

To conclude, we find it absolutely heart warming when a patient is sedated at home and has the opportunity to close their eyes there for the last time, before being transported to the hospital where they are allowed to die peacefully and even help other patients in need. However, this type of procedure should be promulgated with caution, as there are still several logistical, financial, ethical and legal aspects to be discussed.

## Data Availability

Not applicable.

## Abbreviations

MAiD: medical assistance in dying
ODEH: organ donation after euthanasia starting from home
